# Coping with climate change: The role of climate related stressors in affecting the mental health of young people in Mexico

**DOI:** 10.1371/journal.pgph.0002219

**Published:** 2023-09-27

**Authors:** Jessie Pinchoff, Ricardo Regules, Ana C. Gomez-Ugarte, Tara F. Abularrage, Ietza Bojorquez-Chapela

**Affiliations:** 1 Population Council, New York, NY, United States of America; 2 Population Council, Mexico City, Mexico; 3 Max Planck Institute for Demographic Research, Rostock, Germany; 4 El Colegio de la Frontera Norte, Tijuana, Mexico; Conservatoire national des arts et metiers, FRANCE

## Abstract

Young people today are predicted to experience more climate change related stressors and harms than the previous generation, yet they are often excluded from climate research, policy, and advocacy. Increasingly, this exposure is associated with experience of common mental health disorders (CMD). The VoCes-19 study collected surveys from 168,407 young people across Mexico (ages 15–24 years) through an innovative online platform, collecting information on various characteristics including CMD and experience of recent climate harms. Logistic regression models were fit to explore characteristics associated with CMD. Structural equation models were fit to explore pathways between exposure, feeling of concern about climate change, and a sense of agency (meaning the respondent felt they could help address the climate crisis) and how these relate to CMD. Of the respondents, 42% (n = 50,682) were categorized as experiencing CMD, higher among those who experienced a climate stressor (51%, n = 4,808) vs those not experiencing climate stressors (41%, n = 43,872). Adjusting for key demographic characteristics, exposure to any climate event increased the odds of CMD by 50% (Odd Ratio = 1.57; 95% Confidence Interval (CI) 1.49, 1.64), highest for heatwaves. Specific climate impacts such as housing damage, loss of or inability to work, damage to family business, leaving school and physical health affected were adversely related to CMD, though for different climate hazards. More concern and less agency were related to CMD through different pathways, particularly for those exposed to recent events. Future research regarding the cumulative exposures to climate change, not just acute events but as an ongoing crisis, and various pathways that influence the mental health and well-being of young people must be clearly understood to develop programs and policies to protect the next generation.

## Background

The climate crisis is accelerating, with people today already experiencing increasing frequency and intensity of events such as droughts, hurricanes, heatwaves and extreme rainfall [1]. Exposure to these events creates uncertainty, loss, and current and future threats that can produce distress and affect mental health and wellbeing [1–3]. The climate crisis particularly threatens the rising generation of young people, as children born in 2020 are estimated to experience a two- to seven-fold increase in extreme events, particularly heat waves, compared to people born in 1960 [4]. However, young people continue to be excluded from research and policy regarding climate change, although this is starting to change with growing youth activist movements [5–8].

The possible mental health effects of climate change are multifaceted, including post-traumatic stress disorder, depression, anxiety, and suicide [9]. Mental health consequences of climate change can be a direct result of trauma suffered due to climate events including wildfires, hurricanes, and droughts [10, 11]. Indirect consequences of climate change including loss of land, migration, exposure to violence, food insecurity, and changes in ecological, economic, and cultural environment leading to the disintegration of cultural ties between people and their lands that all may also impact and interact with mental health [12, 13] Additionally, emotional impacts of climate change may manifest through dread of environmental disaster, climate anxiety and dissatisfaction with government responses and inaction [11, 12, 14]. Climate change-induced events, particularly heatwaves, may also exacerbate existing acute and chronic mental health conditions [15].

The impacts of climate change will be unequally distributed across populations, with the most vulnerable people and places disproportionately experiencing the most harm. This will intensify existing inequalities including those related to mental health [3, 13, 16]. Evidence consistently shows that specific risk factors including gender, socioeconomic status, education, and pre-existing mental health symptomatology are associated with increased vulnerability to mental health conditions after an extreme weather event [9]. Additionally, living in countries where health systems are not currently providing comprehensive mental health services or where populations are living in poverty, insecurity, or conflict may increase risk of negative mental health outcomes [13].

Children and adolescents are at particular risk for the long lasting and incremental mental and emotional impacts of climate change due to their rapidly developing brains, vulnerability to disease, and limited capacity to avoid or adapt to threats and impacts [12, 14, 16]https://www.zotero.org/google-docs/?vkuY31. According to a recently published global survey, children and young people across all countries were worried about climate change and respondents rated governmental responses to climate change negatively, reporting greater feelings of betrayal than of reassurance [14]. Young people often lack autonomy or control limiting their sense of agency and ability to take up certain coping strategies increasing their concerns and anxieties about the climate crisis [14, 17]. Though poor mental health causes the highest disease burden for youth, with widespread impacts over the trajectory of their lives and even into the next generations, little research focuses on this age group [18, 19]. Compared with adults, relatively little research exists on the mental health effects of climate change and climate-related disasters on children and youth especially when focused on the cumulative exposure and not just on discrete events or disasters but the ongoing crisis [13, 20–22].

Mexico’s geography makes it vulnerable to extreme weather events including hurricanes, floods, droughts, heatwaves, and erratic rainfall [23]. Spatial climate models for this region indicate that, on average, annual temperatures will increase 1.5°C by year 2030, 2.3°C by 2060, and 3.7°C by 2090 [24]. Longitudinal data found that suicide rates rise 2.1% in Mexican municipalities for each 1°C increase in monthly average temperature and recent estimates project that unmitigated climate change could result in a combined 9–40 thousand additional suicides across the United States and Mexico by 2050 [25]. Although Mexico’s population is trending older, as of 2020 still over a quarter (31 million people) are young (15–29 years) and will experience the greatest rise in climate related exposures, with vulnerabilities compounded by continuing inequality driven by poverty and marginalization due to gender and ethnicity [1], additionally facing recent disruptions and harms from the COVID-19 pandemic. Reports cite major gaps in provision of mental health services in Mexico, and adolescents are still not a priority group for federal or state policy-makers [26]. The COVID-19 pandemic spurred the federal Secretary of Health to issue guidelines for mental healthcare but there is still no national defined policy and no focus on young people’s needs [27].

Mexico is working to integrate mental health into a community-based model to increase access to services, yet recent estimates suggest over three-quarters of those with mild, moderate or severe mental disorders still do not receive treatment [28, 29]. Despite Mexico’s new commitments to reducing emissions and transitioning to renewable energy, there are currently no specific policies focused on the effects of climate change on mental health. Climate change should be understood as a social determinant of mental health that will have a strong impact on young people, particularly those from other marginalized groups such as refugees and migrants or indigenous communities facing intersectional risks [10, 12]. Evidence is necessary to inform the development of programs and policies that will adequately meet the mental health needs of Mexico’s diverse young population.

The Violence Outcomes in COVID-19 Era Study (VOCES-19) aimed to reach adolescents and young adults (ages 15–24 years) living in Mexico through an online platform to understand how their lives were impacted by the COVID-19 pandemic and related issues [30]. Questions regarding exposure to climate hazards in the last 12 months, specific harms related to those events and perceptions of climate change were included in the survey conducted in 2022. This paper presents findings from 155,088 young people to understand how recent climate hazards (in the last 12 months) are associated with common mental health disorders (CMD), a combination of reported depression and anxiety symptoms. Further analysis examines how such outcomes are associated with feelings of individual agency and concern related to climate change. Overall, our paper highlights the ways that climate change is affecting adolescent mental health and how this relates to feelings of concern and agency regarding the ongoing climate crisis.

## Methods

The study methods have been described elsewhere [30]. In brief, a longitudinal study was conducted by Population Council Mexico, in collaboration with the National Institute of Youth (IMJUVE) and the National Center for Gender Equity and Reproductive Health. The first survey round was conducted between November 2020 and February 2021, and focused on the potential harms of COVID-19 lockdown policies. In the second wave, conducted from November 2021- June 2022, a module of questions related to climate change was added, as this topic was identified as critical to the lives of adolescents and young people. Each of the two survey waves included a questionnaire that took approximately 35 minutes to complete, and asked a series of questions regarding demographics, exposure to different climate hazards (drought, heatwaves, hurricanes or flooding), related harms such as economic loss or dropping out of school, and also experience of depressive symptoms and anxiety.

### Sampling

The target population for the online survey was adolescents between 15–17 years of age and young adults between 18–24 years living in Mexico. Recruitment occurred via an open invitation through social media and a targeted invitation for youth made by the IMJUVE and different educational authorities [30]. To further disseminate the survey a webpage and social media accounts, radio spots and a press release were also implemented [30]. Participants were required to provide informed consent before filling out the survey. In addition, a second consent was requested to provide their email or cell phone number for future rounds of the study. In Round 1 55,692 completed the survey, in Round 2 168,407 completed the survey (with overlap). The analysis presented in this article includes only those participants of Round 2 who answered the climate hazards questions (n = 152,088). The final database is a large convenience sample of Mexican adolescents. While we acknowledge that the sample was not randomly selected and therefore not representative of the wider population, statistical methods were used to adjust for key demographic characteristics. Although not fully accounting for potential biases, the results provide valuable insights into the perspectives and experiences of a large and diverse group of young people in Mexico, highlighting key focus areas for future research.

### Data analysis

Three mental health outcome variables were explored. First, depressive symptoms were assessed with the Patient Health Questionnaire (PHQ-9) [31], an instrument that is considered a strong diagnostic indicator for detecting depressive disorders for cut-off scores between 8 and 11 [32]. The PHQ-9 was categorized into a binary variable, with 9 serving as the cut off for depressive symptoms [32]. Second, symptoms of anxiety were assessed with the Generalized Anxiety Disorder (GAD) questionnaire, which detects anxiety with a score cut off of 10 (89% sensitivity and 82% specificity) for moderate or severe anxiety [33]. Finally, participants scoring over the cutoff of either instrument were considered to show symptoms of a common mental health disorder (CMD).

Control variable for the analysis included gender (male, female, trans or non-binary), a two-level age variable (15–17 vs 18–24 years), a 4-category combination of working or in school (in school only, study and working, not studying), a five-category region variable (central, south, central north, north west, or north), whether the respondent resided in an urban or rural municipality, wealth tertile (calculated for each respondent with three levels, low, medium and high wealth), and household composition (living with: 1) both parents, 2) one parent only, 3) neither parent but with family, 4) with spouse and children if any, and 5) other includes living alone, with roommates, or other living situations). Wealth tertile was calculated regressing per-capita income against a set of household and dwelling variables in the Mexican National Survey of Income and Expenditure (ENIGH for its acronym in Spanish) [34]. Then we used the regression coefficients and the household and dwelling characteristics of the VOCES-19 participants to impute their wealth tertile.

Two variables were used to define exposure to a climate event, first a binary response to the question “in the last 12 months did you experience any climate related event?”, and then the follow up response to “which event impacted you the most” of which respondents could select flooding, hurricane, heat wave or drought. Respondents were then asked how they were impacted (and could select more than one): damage to the home, lost work or could not work, harm to the family business, left school, damage to crops or livestock, or severe health impacts.

Lastly, structural equation models (SEM) were conducted for path analysis to understand how exposure to a climate event related to concern about climate and feelings of agency about the crisis. To measure this concern, participants responded to the question “when you think about the future, how much do you think climate change will affect you?”, responses were none, a little, a lot, or no response. To measure agency, participants were asked “what do you think you can do to help solve climate change?”. Responses were: nothing—it’s very complicated, a little—what I can do to help won’t help much, something—every action adds up, a lot—if we all organize we can address the issue. The SEM model explored how CMD related to concerns and agency adjusting for demographic characteristics including age, gender, education and work, household composition, wealth tertile, rural vs urban, and region of residence. It was hypothesized that those who were exposed to a climate event would feel more concern, and experience CMD, though this might be moderated if the respondent felt more agency to do something about climate change.

### Ethics

The study received institutional review board (IRB) approval from the Population Council (IRB Research Protocol No. 949). All participants aged 18–24 years old provided informed consent by filling in an electronic form displayed before the survey started. Since the online study presented minimal risks to participants for those aged 15–17 years old, parental consent waivers were granted. The online questionnaire did display an assent form to inform minors on the aim, risks, and benefits of the study, but it was deemed that the benefits outweighed the risks. Participation was restricted to respondents who provided assent (15–17 year olds) or consent (18–24 year olds). The STROBE checklist is included as a supplement (S1 Checklist) and the PLoS Global Inclusivity Statement (S1 Text).

### Funding

An anonymous donor provided funding for the study to JP. The donor had no role in study conceptualization, study design, data collection and analysis, the decision to publish, or preparation of the manuscript.

## Results

Of all respondents, over one third (n = 42%; 48,680) were categorized as having CMD. There were no differences by age with respect to CMD, but slight differences by gender, household composition, wealth tertile, in school or working, region and urban or rural residence were noted in descriptive comparisons (**Table 1**). Overall, 8.1% of respondents experienced a climate related hazard in the last 12 months, mainly floods followed by hurricanes, heatwaves, and drought (**Table 2**). CMD was highest for those who experienced a heat wave (57%), flood (52%), drought (51%) and hurricane (47%) (**Table 2**).

**Table 1:** Descriptive characteristics of respondents comparing those who did and did not experience CMD.

	No CMD	CMD	Total	p-value
	N = 67,332	N = 48,680	N = 116,012	
15–17 years	58,723 (87%)	42,516 (87%)	101,239 (87%)	0.53
18–24 years	8,609 (13%)	6,164 (13%)	14,773 (13%)	
Gender				
Male	32,352 (48%)	16,722 (29%)	46,074 (40%)	<0.001
Female	33,742 (51%)	32,000 (67%)	65,742 (58%)	
Trans/nonbinary	416 (1%)	1,738 (4%)	2,154 (2%)	
Household composition				
Both Parents	47,736 (71%)	31,155 (64%)	78,891 (68%)	<0.001
One parent	16,855 (25%)	15,088 (31%)	31,943 (28%)	
Family, not parents	1,874 (3%)	1,908 (4%)	3,782 (3%)	
Spouse, kids if any	306 (0%)	147 (0%)	453 (0%)	
Alone, roommates, other	267 (0%)	223 (0%)	490 (0%)	
Wealth Tertile				
Lowest tertile	22,914 (34%)	15,804 (33%)	38,718 (33%)	<0.001
Middle	32,593 (48%)	24,010 (49%)	56,603 (49%)	
Highest tertile	11,814 (18%)	8,857 (18%)	20,671 (18%)	
Schooling/Working				
Studying	51,655 (82%)	36,672 (81%)	88,327 (82%)	<0.001
Studying and working	10,692 (17%)	8,194 (18%)	18,886 (17%)	
Not studying	516 (1%)	297 (1%)	813 (1%)	
Region				
Sur	7,874 (12%)	5,955 (12%)	13,829 (12%)	0.016
Centro	46,659 (69%)	33,621 (69%)	80,280 (69%)	
Centro-norte	3,128 (5%)	2,349 (5%)	5,477 (5%)	
Norte-occidente	1,413 (2%)	1,056 (2%)	2,469 (2%)	
Norte	8,258 (12%)	5,699 (12%)	13,957 (12%)	
Urban	53,785 (80%)	40,628 (83%)	94,413 (81%)	<0.001
Rural	13,547 (20%)	8,052 (17%)	21,599 (19%)	
Climate hazard				
Flood	1,607 (35%)	1,714 (36%)	3,321 (36%)	0.85
Drought	581 (13%)	616 (13%)	1,197 (13%)	0.99
Heatwaves	599 (13%)	782 (16%)	1,381 (15%)	<0.001
Hurricane	882 (20%)	801 (17%)	1,690 (18%)	<0.001
Harms from Climate Hazard				
Home damaged	2,109 (47%)	2,321 (49%)	4,430 (48%)	0.09
Lost work/couldn’t work	2540 (6%)	396 (8%)	650 (7%)	<0.001
Damage to family business	222 (5%)	324 (7%)	546 (6%)	<0.001
Dropped out of school	113 (3%)	169 (4%)	282 (3%)	0.004
Damage to crops/livestock	828 (18%)	772 (16%)	1,600 (17%)	0.005
Physical health affected	210 (5%)	451 (9%)	661 (7%)	<0.001

**Table 2:** Characteristics of respondents by type of climate hazard reported.

	No hazard	Any hazard	flood	drought	hurricane	heat	All respondents	p-value
	N = 139,739	N = 12,349	N = 4,209	N = 1,547	N = 2,198	N = 1,857	N = 152,088	
Age 15–17 years	122,198 (87%)	10,594 (86%)	3,517 (84%)	1,299 (84%)	1,943 (88%)	1,612 (87%)	132,792 (87%)	<0.001
Age 18–24 years	17,541 (13%)	1,755 (14%)	692 (16%)	248 (16%)	255 (12%)	245 (13%)	19,296 (13%)	
Male	54,915 (40%)	4,886 (41%)	1,502 (36%)	645 (43%)	997 (46%)	819 (45%)	59,801 (40%)	<0.001
Female	79,585 (58%)	6,910 (57%)	2,506 (61%)	845 (56%)	1,115 (52%)	966 (53%)	86,495 (58%)	
Trans/Nonbinary	2,293 (2%)	257 (2%)	104 (3%)	25 (2%)	41 (2%)	28 (2%)	2,550 (2%)	
Wealth tertile								
Lowest tertile	48,489 (35%)	5,714 (46%)	1,820 (43%)	811 (52%)	1,010 (46%)	901 (49%)	54,203 (36%)	<0.001
Middle	67,517 (48%)	5,237 (42%)	1,897 (45%)	577 (37%)	951 (43%)	773 (42%)	72,754 (48%)	
Highest tertile	23,702 (17%)	1,398 (11%)	492 (12%)	159 (10%)	237 (11%)	183 (10%)	25,100 (17%)	
School/Employment								
Studying	103,821 (82%)	8,251 (74%)	2,890 (75%)	1,009 (72%)	1,472 (75%)	1,171 (70%)	112,072 (81%)	<0.001
Studying and working	21,679 (17%)	2,709 (24%)	916 (24%)	360 (26%)	470 (24%)	473 (28%)	24,388 (18%)	
Not studying	971 (1%)	129 (1%)	36 (1%)	28 (2%)	24 (1%)	23 (1%)	1,100 (1%)	
Who does the respondent live with?								
both Parents	95,317 (69%)	8,201 (66%)	2.719 (65%)	1,102 (72%)	1,496 (68%)	1,236 (67)	103,518 (68%)	<0.001
One parent	38,089 (27%)	3,446 (28%)	1,270 (30%)	345 (22%)	575 (26%)	508 (27%)	41,535 (27%)	
Family, not parents	4,626 (3%)	477 (4%)	147 (3%)	66 (4%)	95 (4%)	76 (4%)	5,103 (3%)	
Spouse, kids if any	531 (0%)	80 (1%)	26 (1%)	14 (1%)	10 (0%)	15 (1%)	611 (0%)	
Alone, roommates, other	572 (0%)	91 (1%)	27 (1%)	12 (1%)	19 (1%)	18 (1%)	663 (0%)	
Region								
Centro	98,668 (71%)	7,041 (57%)	2,753 (65%)	980 (63%)	584 (27%)	1,184 (64%)	105,709 (70%)	<0.001
Sur	15,229 (11%)	3,154 (26%)	815 (19%)	250 (16%)	1,332 (61%)	352 (19%)	18,383 (12%)	
Centro-norte	6,563 (5%)	490 (4%)	135 (3%)	85 (5%)	70 (3%)	86 (5%)	7,053 (5%)	
Norte-occidente	2,796 (2%)	324 (3%)	69 (2%)	48 (3%)	125 (6%)	31 (2%)	3,120 (2%)	
Norte	16,483 (12%)	1,340 (11%)	437 (10%)	184 (12%)	87 (4%)	204 (11%)	17,823 (12%)	
Urban	112,914 (81%)	9,260 (75%)	3,506 (83%)	819 (53%)	1,607 (73%)	1,390 (75%)	122,174 (80%)	<0.001
Rural	26,825 (19%)	3,089 (25%)	703 (17%)	728 (47%)	591 (27%)	467 (25%)	29,914 (20%)	
Mental health outcomes								
Depressive symptoms (PHQ cutoff)	35,522 (34%)	3,820 (43%)	1,351 (43%)	496 (43%)	664 (40%)	601 (46%)	39,342 (35%)	0.011
Anxiety symptoms (GAD cutoff)	31,512 (27%)	3,580 (36%)	1,297 (36%)	477 (37%)	552 (30%)	582 (39%)	35,092 (28%)	<0.001
CMD (yes/no)	43,872 (41%)	4,808 (51%)	1,714 (52%)	616 (51%)	801 (47%)	782 (57%)	48,680 (42%)	<0.001

In fully adjusted logistic regression models, those impacted by any climate hazard in the last 12 months were more likely to have CMD (OR = 1.57, 95% confidence interval (CI): 1.49, 1.64) (**Table 3, Model 1**). Compared to men, women were more likely to report CMD (OR = 2.34, 95% CI: 2.28, 2.40) and trans or nonbinary respondents even more likely to report CMD (OR = 9.90, 95% CI: 8.84, 11.10) (**Table 3, Model 1**). Compared to the poorest tertile, those in the middle or wealthiest tertile were more likely to report CMD. Those residing in the northern region were less likely to report CMD compared to those in the Central region (OR = 0.88, 95% CI 0.85, 0.92). When comparing the effects of specific types of climate events on CMD, the odds of higher CMD were higher for flood, drought, hurricanes and heat waves with drought and heat waves having the highest odds ratios (**Table 3, Model 2**).

**Table 3:** Logistic regression results of characteristics associated with CMD.

	Model 1	Model 2
VARIABLES	OR / (95% CI)	OR / (95% CI)
Impacted by climate in last 12 months	1.565***	
	(1.494–1.640)	
Impacted by (NONE = REF)	NA	REF
Floods		1.492***
		(1.384–1.609)
Drought		1.729***
		(1.527–1.958)
Hurricane		1.352***
		(1.214–1.504)
heatwaves		2.029***
		(1.807–2.279)
18–24 years (vs 15–17 years)	1.003	1.005
	(0.994–1.013)	(0.995–1.015)
Gender (Male)	REF	REF
Female	2.339***	2.344***
	(2.278–2.403)	(2.282–2.408)
Trans/nonbinary	9.902***	10.072***
	(8.837–11.095)	(8.976–11.301)
Urban (vs rural)	1.322***	1.322***
	(1.279–1.367)	(1.288–1.368)
Wealth tertile (Ref = poorest tertile)	REF	REF
Middle tertile	1.117***	1.119***
	(1.085–1.150)	(1.087–1.152)
Wealthiest tertile	1.170***	1.169***
	(1.127–1.215)	(1.126–1.215)
Region (Ref = Central)	REF	REF
South	1.056**	1.056**
	(1.014–1.003)	(1.014–1.100)
Center-North	1.059	1.059
	(0.997–1.125)	(0.997–1.125)
North-West	1.012	1.0117
	(0.927–1.045)	(0.927–1.104)
North	0.881***	0.881***
	(0.846–0.918)	(0.846–0.918)
School/Work (Ref = In school)	REF	REF
Studying and working	1.211***	1.209***
	(1.170–1.252)	(1.168–1.250)
Not studying	0.909	0.908
	(0.771–1.071)	(0.769–1.073)
Household composition (live with both parents = REF)	REF	REF
One parent, only	1.322***	1.326***
	(1.286–1.360)	(1.289–1.364)
Family but neither parent	1.524***	1.520***
	(1.420–1.637)	(1.415–1.633)
Spouse/partner, kids if any	0.549***	0.528***
	(0.441–0.684)	(0.422–0.660)
Other (Alone, with roommates)	1.270*	1.285*
	(1.038–1.553)	(1.048–1.576)
Observations	105,866	104,319

*p<0.05

**p<0.01

***p<0.001

Among those who experienced any climate hazard, there was variation in what climate related harms most affected CMD. Those who experienced housing damage due to a climate hazard had 18% higher odds of CMD compared to those who did not report housing damage (OR = 1.18; 95% CI: 1.07, 1.30) (**Table 4, Model 1**). Those who lost work or could not work due to the climate hazard were also more likely to report CMD compared to those who did not lose work. The most significant climate related harm was physical health. Young people who reported their physical health was harmed due to a climate hazard had over two times higher odds of CMD compared to those who did not report a physical health harm (OR = 2.23, 95% CI 1.86, 2.68) (**Table 4, Model 1**).

**Table 4:** Logistic regression characteristics of factors associated with CMD, stratified by type of climate hazard experienced.

	All hazards	Floods	Droughts	Hurricanes	Heatwaves
VARIABLES	Model 1	Model 2	Model 3	Model 4	Model 5
Climate impact:					
Housing damage (yes vs no)	1.179**	1.219*	1.323	1.145	1.228
	(1.072–1.297)	(1.025–1.449)	(0.905–1.934)	(0.896–1.465)	(0.884–1.706)
Lost or could not work	1.492***	1.254	1.327	1.783**	1.59
	(1.246–1.786)	(0.929–1.691)	(0.815–2.163)	(1.197–2.657)	(0.912–2.773)
Damage to family business	1.452***	0.989	2.119**	1.827*	1.397
	(1.194–1.766)	(0.706–1.386)	(1.368–3.283)	(1.136–2.938)	(0.825–2.365)
Left school	1.234	0.829	2.162	1.824*	1.59
	(0.944–1.613)	(0.555–1.239)	(0.643–7.277)	(1.027–3.239)	(0.666–3.796)
Crop/livestock losses	1.049	0.909	0.956	0.95	1.106
	(0.922–1.193)	(0.676–1.223)	(0.712–1.285)	(0.683–1.323)	(0.814–1.502)
Health affected	2.232***	2.640***	2.962**	1.813	1.992***
	(1.857–2.683)	(1.569–4.441)	(1.486–5.904)	(0.967–3.399)	(1.498–2.648)
18–24 years (vs 15–17 years)	0.982	0.988	0.936	1.069	1.015
	(0.950–1.014)	(0.939–1.039)	(0.857–1.021)	(0.976–1.170)	(0.922–1.117)
Gender (Ref = Male)	REF	REF	REF	REF	REF
Female	2.002***	2.144***	1.596***	2.046***	1.930***
	(1.825–2.196)	(1.830–2.511)	(1.229–2.072)	(1.642–2.549)	(1.518–2.455)
Trans/nonbinary	5.999***	6.383***	26.338**	6.206***	3.154*
	(4.175–8.620)	(3.582–11.374)	(3.481–199.292)	(2.620–14.700)	(1.118–8.897)
Urban (vs rural)	1.358***	1.182	1.486**	1.620***	1.372*
	(1.215–1.517)	(0.957–1.461)	(1.138–1.940)	(1.243–2.111)	(1.030–1.826)
Wealth tertile (Ref = poorest tertile)	REF	REF	REF	REF	REF
Middle tertile	1.029	1.095	0.955	1.166	0.833
	(0.935–1.133)	(0.932–1.286)	(0.727–1.254)	(0.925–1.469)	(0.647–1.071)
Wealthiest tertile	1.172*	1.08	1.121	1.137	1.299
	(1.011–1.358)	(0.845–1.379)	(0.719–1.746)	(0.791–1.633)	(0.862–1.957)
Region (Ref = Centro)	REF	REF	REF	REF	REF
Sur	0.850**	0.84	1.028	0.665**	1.192
	(0.763–0.946)	(0.689–1.022)	(0.729–1.449)	(0.509–0.870)	(0.875–1.623)
Centro-norte	1.320*	1.581*	1.517	0.966	1.173
	(1.044–1.668)	(1.020–2.445)	(0.865–2.662)	(0.506–1.845)	(0.679–2.025)
Norte-occidente	0.712*	0.543	1.262	0.543*	0.783
	(0.542–0.935)	(0.293–1.006)	(0.611–2.605)	(0.334–0.884)	(0.306–2.002)
Norte	0.949	0.749*	0.918	1.507	1.314
	(0.821–1.098)	(0.582–0.963)	(0.617–1.365)	(0.846–2.685)	(0.884–1.953)
School/Work (REF = In school)	REF	REF	REF	REF	REF
Studying and working	1.241***	1.132	1.363*	1.334*	1.215
	(1.113–1.382)	(0.944–1.356)	(1.007–1.846)	(1.064–1.734)	(0.921–1.603)
Not studying	1.426	1.104	4.511*	0.689	3.329
	(0.873–2.331)	(0.457–2.666)	(1.104–18.438)	(0.211–2.512)	(0.826–13.410)
Household composition (both parents = REF)		REF	REF	REF	REF
One parent, only	1.25***	1.358***	1.294	1.359*	1.01
	(1.130–1.382)	(1.154–1.597)	(0.950–1.766)	(1.064–1.735)	(0.773–1.320)
Family but neither parent	1.672***	1.359	1.415	2.532**	1.568
	(1.312–2.135)	(0.894–2.066)	(0.765–2.619)	(1.440–4.452)	(0.834–2.946)
Spouse/partner, kids if any	0.703	1.273	0.375	0.202	0.100**
	(0.403–1.227)	(0.517–3.135)	(0.092–1.529)	(0.021–1.912)	(0.018–0.539)
Other (Alone, with roommates)	0.982	1.534	0.655	0.988	0.839
	(0.559–1.723)	(0.499–4.707)	(0.133–3.222)	(0.300–3.252)	(0.227–3.100)
Observations	8,337	2,996	1,077	1,508	1,237

We also stratified by each specific climate hazard. For those who experienced a flood event in the last 12 months, there was an association between experiencing housing damage and worse CMD compared to those who experienced flood but no housing damage (OR = 1.22, 95% CI: 1.03, 1.45) (**Table 4, Model 2**). Among those who experienced a hurricane, those who lost work or could not work due to the climate hazard had higher odds of CMD compared to those who experienced a hurricane but did not lose work due to the climate hazard (OR = 1.78, 95%: 1.20, 2.66) (**Table 4, Model 4**). Among those who experienced drought, damage to the family business was associated with higher odds of CMD (OR = 2.12; 95% CI: 1.37, 3.28) (**Table 4, Model 3**).

Lastly, the SEM results (**Table 5**) show that concern about climate change or experience of climate anxiety served as a mediator of the association between having been affected by a climate event and CMD. In addition, perceived agency regarding climate change was a moderator of the effect of having been affected by a climate event and concern about climate change. Thus, participants who had been affected by an event, but perceived that they could do something about climate change, were less likely to experience concern about climate, than those who had been affected but perceived there was nothing they could do.

**Table 5:** Mediation and moderation of the association between experiencing a climate event and CMD, by concern about climate change and perceived agency regarding climate change (SEM).

	Coef.	95% CI	p value
Mental health disorders			
Affected by any climate event?	0.44	(0.39, 0.49)	<0.001
Youth (18–24, vs 15–17 years)	-0.03	(-0.07, 0.01)	0.09
Male gender (REF)			
Female gender	0.80	(-0.07, 0.01)	<0.001
Trans/non-binary	2.00	(1.89, 2.10)	<0.001
Education (in school, not working = REF)			
Studying and working	0.19	(0.15, 0.22)	<0.001
Not studying	-0.11	(-0.28, 0.06)	0.20
Household composition (both parents = REF)			
One parent	0.28	(0.25, 0.31)	<0.001
Family, not parents	0.43	(0.35,0.51)	<0.001
Spouse, kids if any	-0.64	(-0.87, -0.42)	<0.001
Alone, roommates, other	0.24	(0.04, 0.45)	0.02
Tertile of wealth (poorest = REF)			
Middle	0.09	(0.07, 0.13)	<0.001
Wealthiest	0.12	(0.08, 0.16)	<0.001
Ubran (vs rural)	0.30	(0.26, 0.33)	<0.001
Region (Central = REF)			
South	0.05	(0.01, 0.09)	0.01
Center-North	0.07	(0.01, 0.13)	0.02
North-West	0.01	(-0.08, 0.10)	0.80
North	-0.08	(-0.12, -0.04)	<0.001
in the future I worry about climate change (none = REF)			
A little	0.25	(0.20, 0.29)	<0.001
A lot	0.61	(0.56, 0.65)	<0.001
_cons	-1.76	(-1.81, -1.70)	<0.001
**in the future I worry about climate change (concern)**			
Affected by any climate event?	0.41	(0.32, 0.50)	<0.001
Actions we can take against climate change? (None, it’s a very complicated topic = REF)			
A little, every bit helps	1.38	(1.34, 1.42)	<0.001
Something, every action adds up	1.71	(1.67, 1.74)	<0.001
A lot, if everyone organizes we can do something about it	2.18	(2.15, 2.22)	<0.001
Affected by any climate event ## actions we can take against climate change			
Yes#A little, every bit helps	-0.45	(-0.58, -0.32)	<0.001
Yes#Something, every action adds up	-0.24	(-0.36, -0.13)	<0.001
Yes#A lot, if everyone organizes we can do something about it	-0.20	(-0.31, -0.08)	0.001
/in the future I worry about climate change (concern)			
cut1	-0.41	(-0.44, -0.38)	
cut2	1.45	(1.42, 1.49)	

## Discussion

Our results highlight that almost one in ten adolescents reported being exposed to a climate event in the last 12 months, a number likely to increase in the coming years. Over a third were classified as having a CMD, higher among those who had experienced a recent climate event. As climate change accelerates it will increasingly impact young peoples’ transitions to adulthood, during an age range that is dense with live events and decisions. Climate related exposures and harms are heterogeneous, more likely to affect the poorest, women and trans youth, and youth who are juggling school and work. This age is vulnerable to both the effects of climate change and to the experience of mental health challenges, and these two interact. Younger generations are increasingly coming up in this broader context of the climate crisis, experiencing chronic concerns about the future and climate anxiety that can also affect mental health.

Mental health costs are the highest single source of global economic burden in the world [35], and adolescence is the peak time for clinical onset of most mental illnesses [21]. Yet access to mental health services are low or uneven in many countries including Mexico, where less than 1% of Mexico’s Ministry of Public Health total budget is dedicated to mental health [27]. Out of pocket expenditures in Mexico have been steadily increasing, reaching 41.3% in 2017, with higher likelihood of catastrophic healthcare expenditures for mental health issues [27]. As a result, issues such as climate change that may drive economic losses and disruptions may exacerbate CMD and also create an additional financial stressor on households. This makes treatment and care difficult if not impossible for many to afford even if available.

Climate change both directly and indirectly impacts mental health. Extreme or chronic events are most likely to impact the most vulnerable youth [36, 37] and climate change acts as a ‘threat amplifier’ exacerbating existing health disparities, including for mental health [6]. However research on climate and mental health specifically for young people is nascent, and more robust data is needed outlining the range of psychological responses in young people, how such responses may be linked to perceived agency to engage in mitigation and adaptation behaviors, and how these factors intersect with mental health outcomes, including symptoms of generalized anxiety and stress [2]. Different climate events may have different adverse impacts and harms that relate to mental health. For example, our study found that flood events were more likely to cause housing damage, leading to higher odds of CMD, while hurricanes were linked with lost work and damage to family businesses that led to higher odds of CMD. Droughts mainly affected mental health through damages to the family business and economic harms, and mostly in rural areas. All four types of climate hazard (drought, floods, hurricanes and heatwaves) led to worse mental health outcomes in particular through an association with physical health. There are strong links between physical and mental health, and exposure to climate risks may impact one or both of these.

Heatwaves were the most associated event with worse CMD, more so than floods or hurricanes. There is a growing body of research on the mental health toll of heatwaves, linked with aggression and GBV, increased emergency room visits for mental health conditions, increased deaths to due to intentional and unintentional injuries, suicide rates [25], likely mediated by financial strain [6]. Studies have also found that extreme heat can impact fetal health and impact birth outcomes and child development, suggesting intergenerational effects if families face exposure. A recent study found that children born in 2020 will experience 2–7 fold increases in climate events, particularly heatwaves, compared to those born in 1960 [4]. While Mexico does have a heat adaptation plan through the Centro Nacional de Prevención de Desastres (CENAPRED), it does not refer specifically to the needs of young people or adolescents.

Many studies on climate treat it as the experience of one-off events instead of part of an ongoing crisis [20]. Young people in particularly are growing distressed, in a recent study of 10,000 young people from ten countries (not including Mexico) over half felt sad, anxious, angry, powerless, helpless and guilty, 45% reported their feelings about climate change negatively affected their daily life and functioning, and three-quarters said they think the future is frightening [14]. Studies show that young people who take part in climate action and report higher pro-environmental behaviors have greater well-being and reduced feelings of being powerless [6, 37]. Coping with the climate crisis can affect engagement and self-efficacy, and taking action can help [6, 38]. Our study in Mexico found that over half of respondents reported being concerned about climate change and the future. While our cross-sectional study cannot establish causality between climate events, CMD, concern, or agency, our results align with literature showing that youth expressing worry or concern over climate, that feel they have less agency, are more likely to have CMD. With support, it is possible these young people can channel their concerns into climate action and policy change while also safeguarding their mental health.

This study has several limitations. First, we reached youth from all over Mexico but it is a sample of young people that responded to the VoCes-19 platform and may not be representative of all young people. Second, the questions are self-reported experiences of anxiety and depressive symptoms that strongly correlate with CMD but are not a measure of clinical diagnosis. Relatedly, experience of a climate hazard is also subjective. Lastly, the survey took place in 2022, after the COVID-19 pandemic. It is likely that some of the CMD is related to the pandemic, although the survey took place over a year after normal activities resumed in Mexico.

In conclusion, our study revealed high rates of CMD in Mexico, with variation by gender, income, geography, and exposure to recent climate hazards. Experiencing a recent climate event significantly and negatively affected mental health, with specific climate harms more likely to affect mental health through distinct pathways. It is important to recognize that climate change is not only about individual weather events, but rather it is a chronic exposure to changing climate and weather patterns that can have a profound effect on various aspects of society, including economic, social and mental health. The Mexican General Law on Climate Change (Ley General de Cambio Climático) recognizes healthcare infrastructure as a key action to mitigate the effects of climate change. However, it is critical to include the specific needs of young people, who make up a quarter of the population and face increasing climate-related harms. To address the negative effects of climate change on mental health, it is imperative to increase access to mental health services and meanwhile promote climate advocacy and policy initiatives, led by or inclusive of young people. These initiatives may support young people to feel empowered to take action and create change, and improve mental health by fostering a sense of agency and community. To ensure action, it is critical to integrate the mental health needs of young people into both climate adaptation and national health plans.

## Supporting information

S1 Checklist Strobe checklist. (DOCX)

S1 Text PLOS inclusivity indicates the inclusivity statement. (DOCX)
